# Flow Regulation in Coronary Vascular Tree: A Model Study

**DOI:** 10.1371/journal.pone.0125778

**Published:** 2015-04-30

**Authors:** Xinzhou Xie, Yuanyuan Wang

**Affiliations:** 1 Department of Electronic Engineering, Fudan University, Shanghai, China; 2 Key Laboratory of Medical Imaging Computing and Computer Assisted Intervention of Shanghai, Shanghai, China; Bascom Palmer Eye Institute, University of Miami School of Medicine;, UNITED STATES

## Abstract

**Background:**

Coronary blood flow can always be matched to the metabolic demand of the myocardium due to the regulation of vasoactive segments. Myocardial compressive forces play an important role in determining coronary blood flow but its impact on flow regulation is still unknown. The purpose of this study was to develop a coronary specified flow regulation model, which can integrate myocardial compressive forces and other identified regulation factors, to further investigate the coronary blood flow regulation behavior.

**Method:**

A theoretical coronary flow regulation model including the myogenic, shear-dependent and metabolic responses was developed. Myocardial compressive forces were included in the modified wall tension model. Shear-dependent response was estimated by using the experimental data from coronary circulation. Capillary density and basal oxygen consumption were specified to corresponding to those in coronary circulation. Zero flow pressure was also modeled by using a simplified capillary model.

**Result:**

Pressure-flow relations predicted by the proposed model are consistent with previous experimental data. The predicted diameter changes in small arteries are in good agreement with experiment observations in adenosine infusion and inhibition of NO synthesis conditions. Results demonstrate that the myocardial compressive forces acting on the vessel wall would extend the auto-regulatory range by decreasing the myogenic tone at the given perfusion pressure.

**Conclusions:**

Myocardial compressive forces had great impact on coronary auto-regulation effect. The proposed model was proved to be consistent with experiment observations and can be employed to investigate the coronary blood flow regulation effect in physiological and pathophysiological conditions.

## Introduction

Coronary circulatory system is responsible for delivering oxygen-rich blood to the myocardium. Coronary blood flow, which is mainly regulated by small arteries and arterioles, can always be matched to the metabolic demand of the myocardium [1–3]. As previously demonstrated, about 70% of coronary vascular resistance is controlled by micro-vessels (<200 μm in diameter) [4]. In these micro-vessels, the vascular smooth muscle (VSM) tone is modulated by local concentrations of vasodilatory metabolites, local hemodynamic factors (intraluminal pressure (myogenic response) and vessel wall shear stress (shear-dependent response)) and conducted response from downstream vessels [5]. Although these controlling factors work throughout the coronary micro-circulation, segmental differences were observed in their relative contributions to vessel tone [6]. With the combination of regional controlling of resistance, coronary circulatory system shows a stable flow over a wide range of perfusion pressure and also an effective adjustment in blood flow in response to altered demand [7, 8].

By characterizing the underlying physiological mechanisms derived from experimental observations, several theoretical models for blood flow regulation were developed. To model myogenic response, direct dependence of diameter or tension generation on intravascular pressure was assumed in early theoretical studies [9, 10]. However, the circumferential stress, rather than the pressure, is the mechanical stimulus more likely to drive the myogenic response [11]. To overcome this problem, a tension-based model for the myogenic response was proposed [12]. For metabolic response, modeling the information transfer mechanism is the crucial aspect [11]. Recently, an evidence showed that the adenosine triphosphate (ATP) released by red blood cells (RBCs) in venules was involved in metabolic flow regulation [13]. Based on this mechanism, Arciero *et al*. developed a theoretical model for metabolic flow regulation [14]. Shear-dependent vasodilation play an important role in blood flow regulation, but relatively few theoretical models have included this effect [15–17]. Based on previous studies, Arciero and Carlson developed a general theoretical model for blood flow regulation [15]. In their model, the myogenic, metabolic and shear-dependent responses were included and the predicted metabolic regulation and auto-regulatory behavior were consist with experimental data from skeletal muscle [15]. However, this model has not been tested with data from the coronary circulation.

Compared with other circulatory systems, coronary circulation has some unique aspects. The contraction and relaxation of the left ventricles will generate myocardial compressive forces which markedly affected the coronary blood flow [7]. Myocardial compressive forces directly act on the vascular beds, and it may have impact on flow regulation by changing the vessel wall tension. Coronary circulation has a very high capillary density (capillary density for cardiac muscle is about 3000–4000/mm^2^, which is much higher than those in skeletal muscle (500–1000/mm^2^)) and a very high basal oxygen consumption (8–10 cm^3^O_2_/(100 cm^3^ min)) [7]. Also, segmental difference in flow regulation mechanism was observed in experiments [17–19]. For these reasons, the theoretical model tested with data from skeletal muscle cannot be directly used in coronary circulation. Development of coronary flow regulation models can date back to the 1990s. Liao and Kuo proposed a diameter-based coronary flow regulation model, which includes only myogenic and shear-dependent responses [17]. By assuming the pressure-diameter relationships in vasoactive segments, Cornelissen *et al*. developed a tension-based coronary flow regulation model, incorporating myogenic, shear-dependent and metabolic responses [16]. However, the myogenic tone was assumed to depend on intravascular pressure in their model. Also, none of these studies took the myocardial compressive forces into consideration. More realistic coronary regulation model is needed to provide further understandings of the underlying mechanisms.

The overall goal of the present study was to analyze the blood flow regulation in coronary circulation. The proposed model includes the unique aspects in coronary circulation. Compressive forces were included in the modified wall tension model. Shear-dependent response was estimated by using the experiment date from coronary circulation. Capillary density and basal oxygen consumption were specified to corresponding to those in coronary circulation. Zero flow pressure (ZFP) was also modeled by using a simplified capillary model. The proposed model can be employed to simulate the regulation of coronary blood flow in physiological and pathophysiological conditions, in order to provide further understandings to improve diagnosis, planning and treatment of cardiovascular diseases.

## Method

### Ethics statement

This work is a simulation study and the ethical approval is not required.

### Model overview

**Fig 1:**
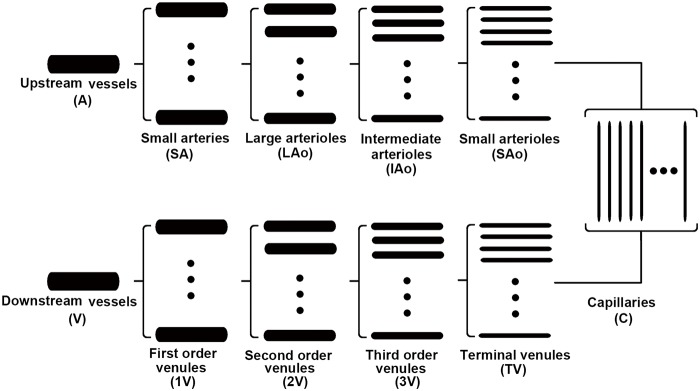
Representative segment model. Coronary vascular tree is represented by 11 regions connected in series. Each region is assumed to contain an array of identical segments in parallel.

A theoretical coronary model was developed to investigate the flow regulation in the coronary vascular tree. It was based on the “representative segment model” proposed by Carlson *et al*. [15]. In their model, the vascular system was represented by 7 regions connected in series, each comprising a set of identical, parallel-arranged segments [15]. According to the amount of experimental data available, the original model was extended to include 11 segments (including four vasoactive segments: small arteries (SA), large arterioles (LAo), intermediate arterioles (IAo) and small arterioles (SAo)) in our coronary vascular tree model as shown in Fig 1. The upstream vessels and venous regions were assumed to have no vasoactive response and modeled as fixed resistances to blood flow. A simplified capillary model was employed to model the zero-flow pressure phenomenon.

### Model for flow regulation

The vasoactive segments are assumed to regulate flow in response to myogenic, shear-dependent and metabolic stimuli [15]. For each segment, the total tension in a vessel wall (*T*_*total*_) is assumed to consist of a passive component (*T*_*pass*_) and an active component (*T*_*act*_):

$$T_{total}=T_{pass}+A\cdot T_{act}^{\max}$$ (1)

where *A* represents the activation characterizing the level of vascular tone [12, 15]. The passive component is given by an exponential function of diameter and the maximal active tension is defined by a Gaussian function of diameter [15]:

$$T_{pass}=C_{pass}\cdot \exp\left[{C^{\prime }}_{pass}\left(\frac{D}{D_{0}}-1\right)\right]$$ (2)

$$T_{act}^{\max}=C_{act}\cdot \exp\left[-{\left(\frac{D/D_{0}-{C^{\prime }}_{act}}{{C^{\prime \prime }}_{act}}\right)}^{2}\right]$$ (3)

where *C*_*pass*_, *C'*_*pass*_, *C*_*act*_, *C'*_*act*_, *C"*_*act*_ are constants and *D*_0_ represents the passive vessel diameter. Values for parameters describing vessel wall mechanics are obtained from the experimental observations and are given in Table 1 [12, 17]. Eqs 1–3 describe the total vessel wall tension based on the length-tension characteristics of vascular smooth muscle [12]. When equilibrium is approached, the amount of total vessel wall tension (Eq 1) would be equal to the vessel wall tension required to keep the vessel wall from rupturing.

**Table 1:** Parameter values for vessel wall mechanics.

	*D*_*0*_ μm	*C*_*myo*_ cm/dyn	*C*_*pass*_ dyn/cm	*C* ^*'*^_*pass*_	*C*_*act*_ dyn/cm	*C'*_*act*_	*C"*_*act*_	C_meta_ μM/cm
Small Arteries (SA)	255.4	0.004674	1719	14.354	1564	0.910	0.374	30
Large Arterioles (LAo)	164.8	0.007508	1141	13.828	1360	0.910	0.374	30
Intermediate Arterioles (IAo)	100.8	0.015977	687	12.606	1131	0.910	0.374	30
Small Arterioles (SAo)	64.9	0.022252	459	13.431	405	0.910	0.374	30

*D*_*0*_, passive vessel diameter; *C*_*myo*_, activation tension sensitivity; *C*_*pass*_ and *C'*_*pass*_, passive tension strength and sensitivity, respectively; *C*_*act*_, *C'*_*act*_, and *C"*_*act*_, maximally active peak tension, length dependence, and tension range, respectively; *C*_*meta*_, activation conducted response sensitivity.

The target activation *A*_*total*_ is given by a sigmoidal function that varies between 0 and 1:

$$A_{total}=\frac{1}{1+\exp\left(-S_{tone}\right)}$$ (4)

where *A*_*total*_ = 0 represents the absence of vascular tone and *A*_*total*_ = 1 represents the maximal vasoconstriction. The level of VSM tone (*S*_*tone*_) representing the net effect of the myogenic, metabolic and shear-dependent responses are defined as:

$$S_{tone}=C_{myo}\cdot T-C_{meta}\cdot S_{CR}-S_{shear}+{C^{\prime \prime }}_{tone}$$ (5)

where *T* is the circumferential wall tension, *S*_*CR*_ is the conducted response signal, *S*_*shear*_ is the shear-dependent response signal, *C*_*myo*_, *C*_*meta*_ and *C"*_*tone*_ are constants [15].

### Modified model for myogenic response

In contrast to other vascular beds, extra forces (myocardial compressive forces) act on the coronary vascular beds [20]. Myocardial compressive forces are generated by the contraction and relaxation of the left ventricles and play an important role in determining the vessel wall tension. The amount of vessel wall tension (*T*) required to keep the vessel wall from rupturing is a product of the transmural pressure (*P*_*tra*_) and the diameter (*D*) of the vessel [21]:

$$T=\frac{P_{tra}\cdot D}{2}$$ (6)

**Fig 2:**
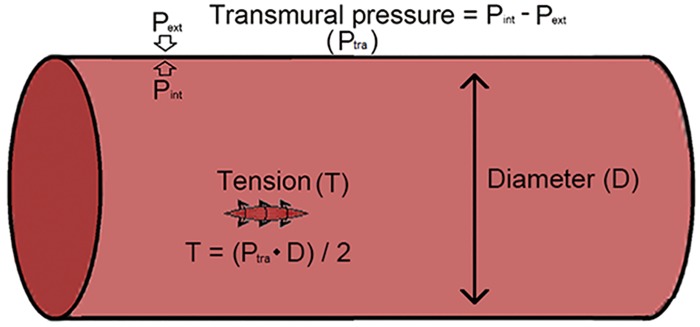
Illustration of transmural pressure, internal pressure, external pressure and vessel wall tension.

The transmural pressure refers to the pressure across the vessel wall and defined as the internal pressure (*P*_*int*_) minus the external pressure (*P*_*ext*_) as illustrated in Fig 2. In most other vascular beds, the external pressure can be ignored due to the very low values and the transmural pressure equals to the internal pressure. However, in coronary vascular beds, the value of the external pressure is related to compressive forces and cannot be ignored. During systole, the compressive force increased much and inducing no-flow or retrograde flow; while during diastole, the compressive force decreased to near zero [22]. Though the compressive force changed rapidly during one cardiac cycle, rate of vascular smooth muscle activation change was much less. Thus, the compressive force was modeled as a steady-state force. In this work, effects of myocardial compressive forces were considered by replacing the internal pressure with the transmural pressure. The external pressure, which is generated due to the myocardial compressive forces, is assumed to be proportional to the perfusion pressure by the constant K. The parameter K was adjusted to give physiologically coronary “pressure-flow” relation.

### Fitted model for shear-dependent response

**Fig 3:**
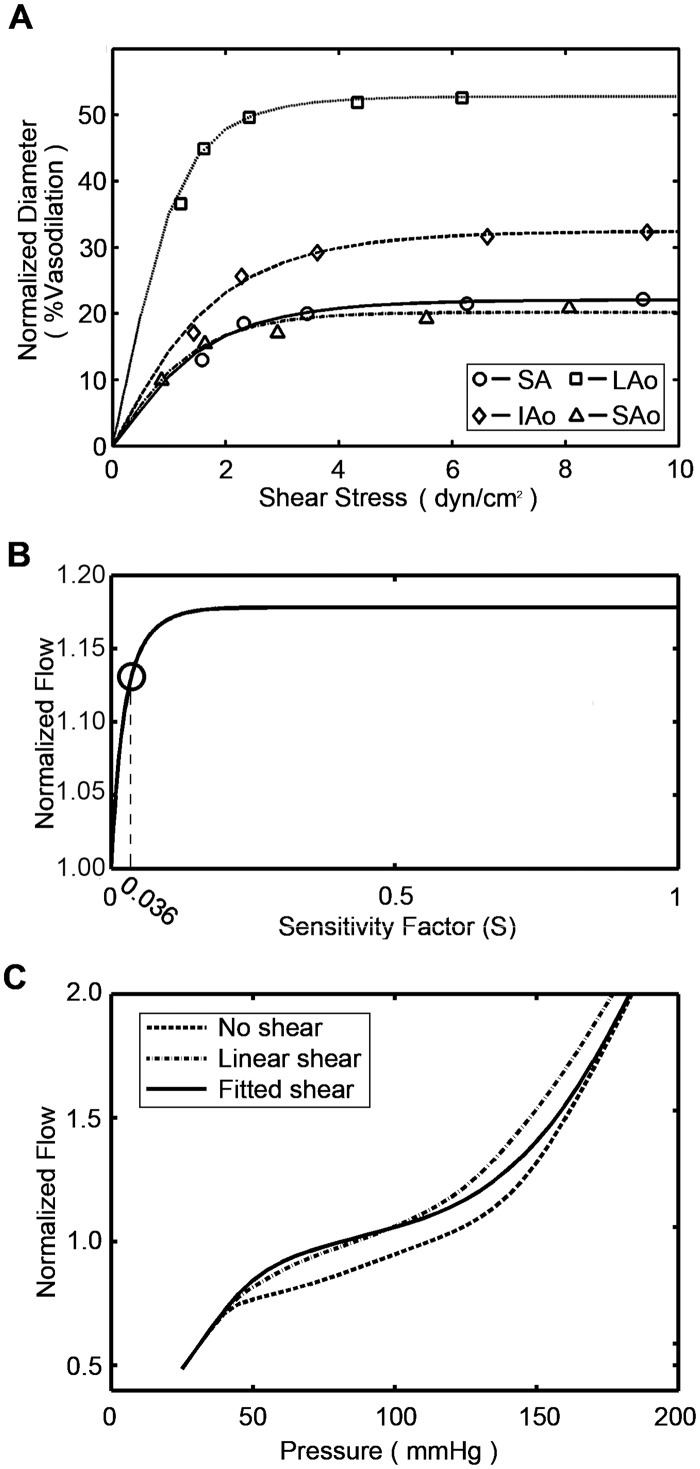
Fitted model for shear-dependent response. (A) Predicted “dilation-shear stress” relations were compared with the experiment data for four vasoactive segments. (B) Effect of sensitivity factor *S* on normalized flow at reference state. (C) Predicted pressure-flow curves without shear-dependent response, with linear shear-dependent response and with fitted shear-dependent response (*S* = 0.036).

In previous model, the shear-dependent response was assumed to be proportional to the wall shear stress [15]. However, experiment observation showed that the shear-dependent response would be much more complex [19]. The vessel “dilation-shear stress” relations for segmental coronary micro-vascular were measured in *vitro* by Kuo *et al*. [19]. In this work, data of Kuo *et al*. were used to estimate the shear-dependent response of four vasoactive segments. First, the shear-dependent response signals were recalculated from the original dilation response by using the gradient descent method. Then, the shear-dependent response signals were fitted by an exponential function of the shear stress:

$$S_{shear}=a\cdot \left[\exp(-b\cdot \tau_{wall})-1\right]$$ (7)

where *τ*_*wall*_ is wall shear stress, *a* and *b* are constant parameters. Values for parameters governing shear-dependent response signals were given in Table 2. The resultant “dilation-shear stress” relations are consistent with the experiment data in *vitro* as shown in (Fig 3A). But, shear stress sensitivity is different in *vivo* from that in *vitro*. Kuo *et al*. reported that values of the shear stress at 2 to 4 dyn/cm^2^ produced maximal vasodilation in isolated coronary arterioles [19]. This value was much smaller than those obtained in *vivo* [23]. The reasonable explanation could be that hemoglobin in blood (in *vivo*) scavenges NO, it may make the shear stress sensitivity become lower in *vivo* [23, 24]. Cornelissen *et al*. introduced an attenuation factor to fill the gaps between the observations in *vitro* and in *vivo* [16]. Here, a similar method was used and a sensitivity factor *S* was introduced:

$$S_{shear}=a\cdot \left[\exp\left(-b\cdot \left(S\cdot \tau_{wall}\right)\right)-1\right]$$ (8)

**Table 2:** Parameters values for shear-dependent responses.

	Small Arteries (SA)	Large Arterioles (LAo)	Intermediate Arterioles (IAo)	Small Arterioles (SAo)
a	-1.519	-2.794	-1.726	-1.035
b	0.6729	1.008	0.615	0.969

The value of *S* varies between 0 and 1. The effect of sensitivity factor *S* on the normalized flow at reference state was shown in (Fig 3B). To make sure that the shear-dependent mechanism is effective, *S* was chosen to be 0.036. (Fig 3C) gave the pressure-flow lines without shear-dependent response, with linear shear-dependent response and fitted shear-dependent response (S = 0.036).

### Model for oxygen transport and metabolic response

Oxygen transport was assumed to occur in arteriolar and capillary segments according to the model proposed by Arciero *et al*. [14]. For each oxygen-delivering vessel, the Krogh-type cylinder model was used to represent the tissue region to which it is exclusively responsible for supplying oxygen [14]. The width of the tissue sleeve was assumed to be 11.8 μm and kept the same for all oxygen-delivering vessels, resulted in a capillary density of 3000/mm^2^. Conducted metabolic signals (*S*_*CR*_) were assumed to be ATP concentration dependent and generated in all segments [14]. A detailed description of oxygen transport and metabolic response can be found in reference 14 and related parameter values were given in Table 3.

**Table 3:** Parameter values for oxygen transport and metabolic response.

Description	Parameter	Value	Unit
Oxygen demand	*M*_*0*_	8–60	cm^3^ O_2_·100 cm^-3^·min^-1^
Oxygen capacity of RBCs	*c*_*0*_	0.5	cm^3^ O_2_/cm^3^
Tube hematocrit	*H*_*T*_	0.3	/
Discharge hematocrit	*H*_*D*_	0.4	/
Maximal rate of ATP release	*R*_*0*_	1.4*10^^-6^	mol·s^-1^/L
Effect of S(x) on ATP release	*R*_*1*_	0.891	/
Initial oxygen saturation	*S*(*0*)	0.97	/
Initial ATP concentration	*C*(*0*)	0.5	μM
Rate of ATP degradation	*k*_*d*_	2*10^^-4^	cm/s
Length constant for S_CR_	*L*_*0*_	1	cm

RBC, red blood cell; ATP, Adenosine Triphosphate; *S*(*x*), oxygen saturation; S_CR_, conducted response signal.

### Model for zero-flow pressure

Coronary zero-flow pressure (ZFP) is the pressure at zero coronary flow. Extra pressure is required to force the slightly oversized red cells through the small blood vessels, leading to that coronary blood flow would reduce to zeros before the coronary pressure gradient reduces to zeros [25]. In our model, this effect was simply modeled by a pressure-related capillary model. The diameter of the capillary (*D*) was assumed to be related to the mean pressure of the capillary (*P*):

$$\{\begin{array}{c} D=\frac{P}{P_{ZFP}}D_{0}\;\;\;\;if\;\;\;\;P<P_{ZFP}\\ D=D_{0}\;\;\;\;if\;\;\;\;P\geq P_{ZEP} \end{array}$$ (9)

where *D*_*0*_ is the diameter in reference state, *P*_*ZFP*_ is the zero-flow pressure. When the mean pressure of capillary is decreasing, the decrease in diameter leads to strong increase in resistance, causing that the coronary blood flow quickly reduces to near zero. The value of *P*_*ZFP*_ was chosen to be consistent with the measured “pressure-flow” relation [26].

### Reference state

The reference state for this coronary vascular tree model was chosen to correspond to resting condition. Diameters for four vasoactive segments (in reference state) were specified to consistent with the experimental observations of Kuo *et al*. [17]. Parameters for each region (the vessel length, the vessel number and the flow rate) were determined by specifying proper wall shear stresses and pressure drops [15]. The pressure drop for upstream vessels was assumed to be 13 mmHg and that for downstream vessels was specified to result in a total perfusion pressure of 100 mmHg in reference state. With the assumption that the vascular network is symmetric (the artery regions have the same number and length as those of the venous regions), the unspecified values of length, diameter, number of vessels and pressure drop can be calculated for each region [15]. Equilibrium is assumed in reference state, and so that *T* = *T*_*total*_ and *A* = *A*_*total*_. Then, the values of *C"*_*tone*_ for each vasoactive compartment can be determined in reference state based on Eqs (1) and (4). The detailed parameters for each segment were shown in Table 4.

**Table 4:** Values of structural and hemodynamic parameters in the reference state.

Parameter	SA	LAo	IAo	SAo	C	TV	3V	2V	1V
Diameter, *D*, μm	**179**	**98**	**64**	**37**	**6**	65.2	116.7	178.6	323.4
Wall shear stress, *τ*, dyn/cm^2^	**55**	**55**	**55**	**55**	**55**	**10**	**10**	**10**	**10**
Pressure drop, *ΔP*, mmHg	**13**	**13**	**13**	**13**	**20**	1.34	1.30	1.30	1.31
Number of segments, *n*	**1**	5.51	18.46	96.19	96386	96.19	18.46	5.51	1
Segment length, *L*, cm	1.41	0.77	0.50	0.29	0.073	0.29	0.50	0.77	1.41
Viscosity, *μ*, cP	2.49	2.26	2.10	2.12	9.05	2.11	2.33	2.49	2.67

Values in bold are prescribed, and the remaining values are calculated based on geometric and hemodynamic considerations.

### Simulation procedure

A dynamic representation method was employed to approach the steady-state conditions for four vasoactive segments [14]. The mean perfusion pressure varied from 25 to 195 mmHg, in order to predict pressure-flow relationship under the assumption of the proposed model. The corresponding flow through the vessel segments was normalized to its value in reference state. The effect of oxygen demand on auto regulation was investigated. Metabolic dilation for adenosine infusion and inhibition of NO synthesis were also simulated and compared with experimental data, to further validate the proposed model. By inserting a stenosis section in upstream vessel, flow regulation in blocked coronary during moderate exercise condition (M_0_ = 30 cm^3^O_2_/(100 cm^3^ min)) was also investigated.

## Results

**Fig 4:**
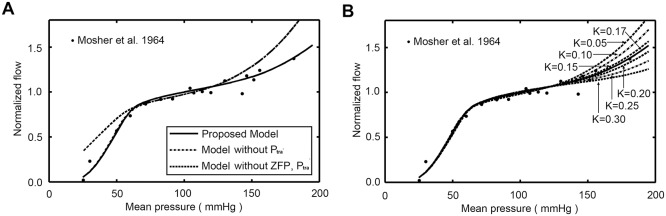
Model predicted pressure-flow curves. (A) Pressure-flow relations predicted by models with and without myocardial compression effect and zero-flow pressure model are compared with experimental data. (B) The effect of parameter K on predicted pressure-flow relation.

In (Fig 4A), model predictions are compared with experimental data [26]. The pressure-flow relations predicted by the proposed model are consistent with experimental data in rest condition. If the myocardial compression effect was not included, the auto-regulatory range would become narrowed and the predicted flow rate would be much higher than experiment observation at high pressure region. If the model for zero-flow pressure was not included, the predicted flow rate would be much higher than experiment observation at low pressure region.

The effect of parameter K on predicted pressure-flow relation was shown in (Fig 4B). Results clearly showed that the myocardial compressive forces had great impact on coronary auto-regulation effect. A larger value of K represented a larger impact of myocardial compressive forces, which resulted in a larger value of external pressure. The auto-regulatory range would be extended if a larger value of K was employed. To make sure the proposed model gives physiologically coronary pressure-flow relationship, K was set for further analyses to 0.17.

**Fig 5:**
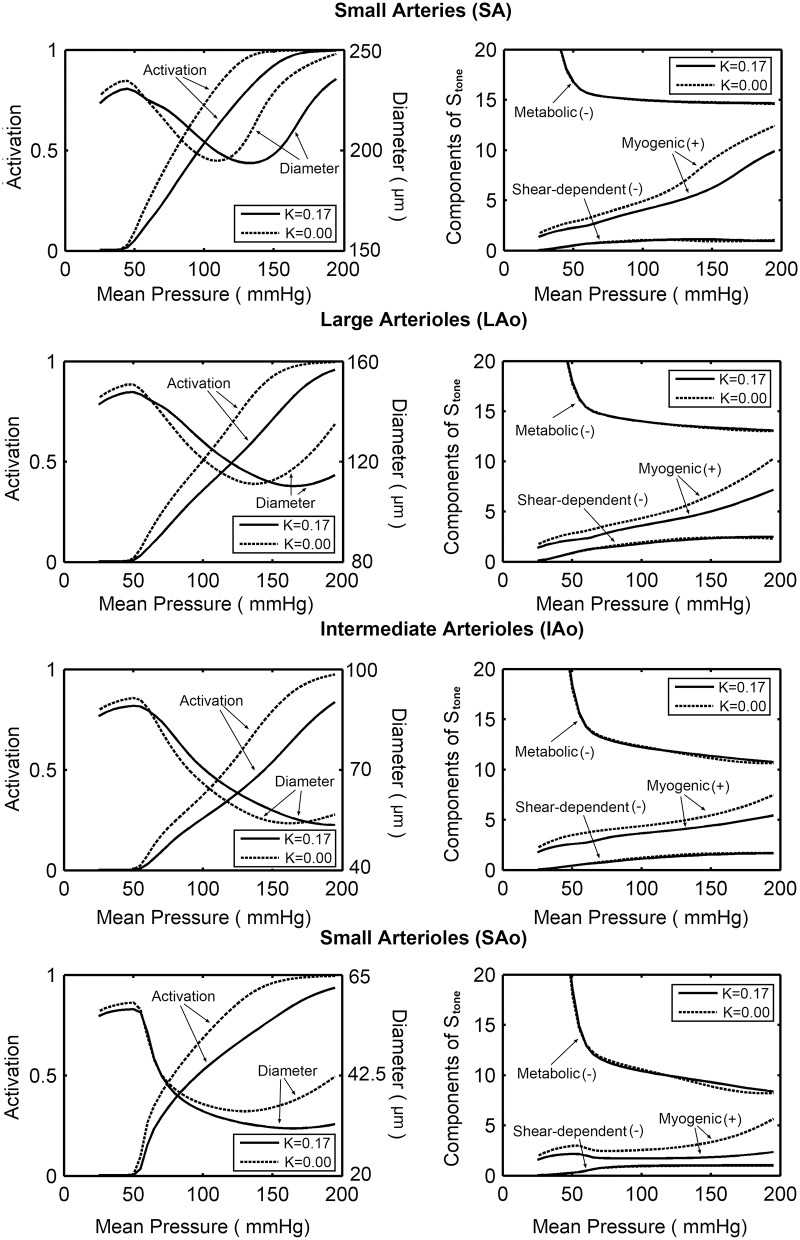
Model predicted blood flow regulation behaviors. (A) Predicted activations and diameters as functions of mean pressure in the four vasoactive segments with and without considering the compressive forces. (B) Contributions of the response mechanisms to the changes in vascular tone with and without considering the compressive forces.

Fig 5 (Left) showed model predicted activations and diameters as functions of the mean pressure in four vasoactive segments with and without considering the compressive forces. At a given pressure, the activations for model with the compressive forces were lower than those for model without it. For model with the compressive forces, as the mean pressure increasing, the activation increases for all segments. The SA would be more activity than the downstream segments. Before the full activation is reached in SA, the diameter would decrease with the increasing pressure. But, once the full activation is reached, the diameter would increase with the increasing pressure. Fig 5 (Right) showed the contributions of the response mechanisms to the changes in vascular tone. The compressive force leads to significant decrease in myogenic tone, but has minor impact on shear-dependent and metabolic tone. The myogenic responses played a more important role in upstream segments than those in SAo. As the pressure increases, the metabolic tone decreased more rapidly in downstream segments. The shear-dependent tone would increase with the increasing pressure in all vasoactive segments, but would contribute more in LAo than in other segments.

**Fig 6:**
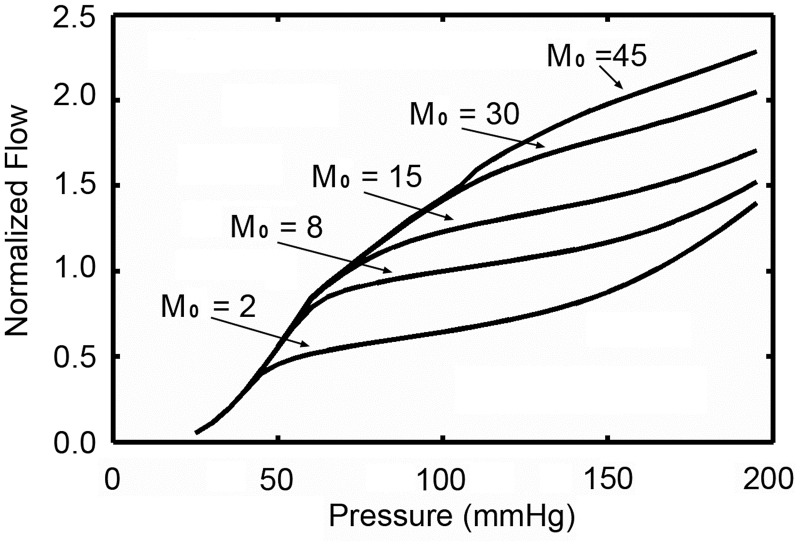
The effect of oxygen demand on auto-regulation behavior.

The effect of oxygen demand on auto-regulation behavior was shown in Fig 6. For a given pressure, the coronary blood flow increases with the increase of oxygen demand. The general shape of auto-regulation curve was preserved, but the auto-regulatory range would be narrowed with the increase of oxygen demand. When the oxygen demand reaches to 45 cm^3^O_2_/(100 cm^3^ min), the auto-regulatory behavior is lost.

**Fig 7:**
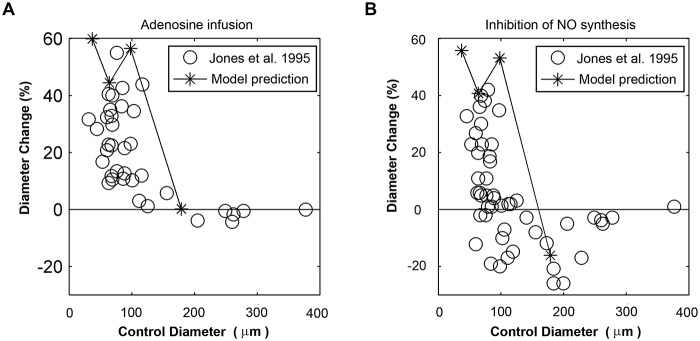
Predicted diameter changes in adenosine infusion and inhibition of NO synthesis conditions were compared with experimental data. (A) Diameter changes in adenosine infusion. (B) Diameter changes in inhibition of NO synthesis.

Adenosine infusion will lead to metabolic dilation in micro-vessels and the responsiveness to adenosine gradually decreases as vessel size increase [17]. Here, metabolic dilation caused by adenosine was simulated by reducing the activations of arterioles to zeros [27]. Inhibition of NO was simulated by setting shear-dependent tone to zeros and reducing the activations of arterioles to zeros [27]. The comparisons between predicted diameter changes and experiment observations were shown in Fig 7. Distal dilation caused by adenosine leads to the increase of flow and decrease of the mean pressure (from 81.5 mmHg to 71.4 mmHg) for SA. Activation of SA was decreased from 0.64 to 0.55, causing that the diameter of SA almost unchanged even with the decreasing mean pressure. Inhibition of NO synthesis leads to a decrease in diameter with increasing activation (from 0.64 to 0.76) for SA. Due to the constriction of SA, the flow increased only 12% of the maximal dilated values. These results were in fair agreement with the observation of Jones *et al*, that no significant changes in blood flow were found by inhibition of NO synthesis [27].

**Fig 8:**
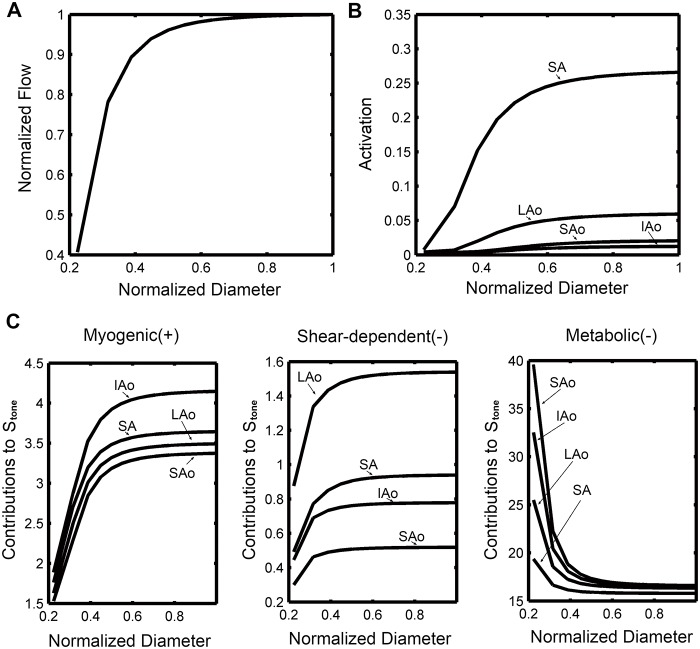
Flow regulation behavior in blocked coronary during moderate exercise condition. (A) Normalized flow rate. (B) Activation. (C) Contributions of the response mechanisms. (Diameters of stenosis section were normalized to its value in reference state (without stenosis)).

Fig 8 showed the flow regulation behavior as functions of the diameter of stenosis section. During moderate exercise condition, flow rate began to decrease as the diameter of upstream stenosis section reducing to about 70% of the normal level. Flow rate reduced to about half of the normal level when the diameter of stenosis section reduced to about 25%. Flow regulation behavior was quite different among the four vasoactive segments. During moderate exercise condition, the activation level of arterioles were quite low; the activation of SA was about 0.27 without stenosis and it reduced rapidly as the flow rate began to reduce. IAo had a larger myogenic response and LAo had a larger shear-dependent response. As the flow rate decreased, metabolic responses for these micro-vessels increased rapidly, while the myogenic and shear-dependent responses decreased.

## Discussion

A theoretical model for coronary blood flow regulation was presented in this paper. Coronary segmental difference in flow regulation mechanism was considered in our model based on previous experimental observations. The impact of myocardial compressive forces on coronary flow auto-regulation behavior was investigated. The predicted pressure-flow curves showed a good agreement with experiment measurements. Adenosine infusion and inhibition of NO synthesis were simulated and the predicted diameter changes in small arteries were in fair agreement with previous observations. The proposed model can be employed to simulate coronary flow regulation in physiological and pathophysiological conditions.

Two previous model studies have concentrated on coronary flow regulation [16, 17]. However, both of these two models expressed the myogenic response as a function of intravascular pressure. Carlson *et al*. proposed a general regulation model in which the myogenic tone is assumed to depend on wall tension. But coronary circulation has some unique aspects and the model cannot be directly employed. Myocardial compressive forces have great impact on coronary flow regulation, but none of these models include this effect. Our proposed model was based on previous “general model”, and the compressive forces were considered by using a modified myogenic model. Impact of the compressive forces was investigated and results showed that the compressive forces acting on the vessel wall will extend the auto-regulatory range by decreasing the myogenic tone at the given perfusion pressure. Also, shear-dependent responses for four different coronary segments was fitted with experiment data and incorporated into the proposed model.

The presented model can be employed to investigate the regulation effect in physiological and pathophysiological conditions. Coronary circulation has a good auto-regulation between 60 and 200 mmHg perfusion pressures. In high blood pressure (hypertension) condition, the coronary resistances will increase to maintain the normal flow rate by increasing the activation of vasoactive segments. The increase of myogenic tone contributes much to this regulation behavior. However, this work demonstrated that due to the increase of compressive forces in high blood pressure condition, the changes of myogenic tone might be overestimated. By inserting extra flow resistances in the model tree, the blocked coronary circulation can be easily modeled with proposed model and the modeling approaches can contribute to the understanding of detailed regulation behavior in these blocked conditions. Stenosis in upstream vessel would block coronary flow and this phenomenon would be more pronounced during exercise condition. From results, the regulation behaviors for different micro-vessels were quite different in blocked coronary system during moderate exercise condition. When flow rate decreased, the oxygen demand could not be matched, and this resulted in a rapidly increase of metabolic responses.

We have to clarify that this study has some limitations. A highly simplified coronary vascular tree model consisting only 11 representative segments was employed in this study, which excludes the complex structure of arterioles. Our model characterized the overall features of coronary flow regulation, and we did not make a distinction between epicardial and endocardial flow. Compressive force played a more important role in subendocardial flow due to the extra flow resistances generated by contraction. Inserting contraction-induced resistance in current model would be a feasible method to model it. It was proven that the external pressure (intramyocardial pressure) depended on mechanical cross-talk [22]. However, it was difficult to give an accurate expression to describe it due to the lack of experimental data. Thus, in this work, a widely accepted assumption was employed and the external pressure (intramyocardial pressure) was assumed to be based on perfusion pressure. A constant capillary density was assumed in present model. However, with increasing metabolic demand, the number of capillaries containing moving red blood cells would increase (capillary recruitment). Mechanism for capillary recruitment has been unclear and was excluded in this model [28]. Also, diffusive interactions between neighboring vessels were not included by using the simplified oxygen transport model. In this work, the venous regions were assumed to have no vasoactive response and modeled as fixed resistances to blood flow. In reality, the diameter of venous may vary with arterial pressure, but the resistances of these vessels are relatively small compared to the total flow resistance. So the effects of these vessels on auto-regulation were neglected. ZFP was thought to be caused by arteriolar tone and/or tissue pressure surrounding the vasculature [29, 30]. Multiple parallel channels also likely affected ZFP. These made modeling ZFP by a pressure-related capillary model imprecise. However, this work concentrated on coronary flow regulation and the capillary region was assumed to have no vasoactive response. In fact, the capillary and venous regions were included in this model, in order to allow simulation of conducted responses. Thus, this simplification would have less impact on the simulated results. Finally, due to lack of detailed underlying mechanism, the model could only predict results in steady-state condition. The pulsating behavior of coronary flow made coronary circulation a very dynamic one. Heart rate dictated the relative systolic and diastolic cycle time, and further had an effect on pulsating flow, pulsating pressure and pulsating shear stress. The pulsating behavior of coronary flow would have an effect on vascular tone, and should be further investigated.

In conclusion, this paper presented a coronary specific blood flow regulation model. Myogenic, shear-dependent and metabolic responses were involved in this regulation model. Specific aspects for coronary circulation, such as myocardial compressive forces, zero-flow pressure, high capillary density and high basal oxygen consumption, were considered in this model. Segmental difference in flow regulation mechanism was included based on previous experimental observations. The model predictions showed good agreement with experiment data and was proved to have abilities to simulate the regulation of coronary blood flow in physiological and pathophysiological conditions.

## References

[1] Chilian WM, Eastham CL, Marcus ML. Microvascular distribution of coronary vascular resistance in beating left ventricle. Am J Physiol. 1986; 251: 779–788. 10.1152/ajpheart.1986.251.4.H779 3766755

[2] Muller JM, Davis MJ, Chilian WM. Integrated regulation of pressure and flow in the coronary microcirculation. Cardiovascular research. 1996; 32: 668–678. 8915185

[3] Knaapen P, Camici PG, Marques KM, Nijveldt R, Bax JJ, Westerhof N, et al Coronary microvascular resistance: methods for its quantification in humans. Basic research in cardiology. 2009; 104: 485–498. 10.1007/s00395-009-0037-z 19468781 PMC2722717

[4] Chilian W, Layne S, Nellis S. Microvascular Pressure Profiles in the Left and Right Coronary Circulations In: Kajiya F, Klassen G, Spaan JE, Hoffman JE, editors. Coronary Circulation: Springer Japan; 1990 p. 173–187.

[5] Segal SS. Regulation of blood flow in the microcirculation. Microcirculation. 2005; 12: 33–45. 15804972 10.1080/10739680590895028

[6] Jones CH, Kuo L, Davis M, Chilian W. Distribution and Control of Coronary Microvascular Resistance In: Sideman S, Beyar R, editors. Interactive Phenomena in the Cardiac System. Advances in Experimental Medicine and Biology. 346: Springer US; 1993 p. 181–188. 7910430 10.1007/978-1-4615-2946-0_17

[7] Duncker DJ, Bache RJ. Regulation of coronary blood flow during exercise. Physiological reviews. 2008; 88: 1009–1086. 10.1152/physrev.00045.2006 18626066

[8] Tune JD, Gorman MW, Feigl EO. Matching coronary blood flow to myocardial oxygen consumption. Journal of applied physiology. 2004; 97: 404–415. 15220323 10.1152/japplphysiol.01345.2003

[9] Feldberg R, Colding-Jorgensen M, Holstein-Rathlou N. Analysis of interaction between TGF and the myogenic response in renal blood flow autoregulation. American Journal of Physiology-Renal Physiology. 1995; 269: 581–593. 10.1152/ajprenal.1995.269.4.F581 7485545

[10] Gonzalez-Fernandez JM, Ermentrout B. On the origin and dynamics of the vasomotion of small arteries. Mathematical biosciences. 1994; 119: 127–167. 8142694 10.1016/0025-5564(94)90074-4

[11] Secomb TW. Theoretical models for regulation of blood flow. Microcirculation. 2008; 15: 765–775. 10.1080/10739680802350112 18951240 PMC2593747

[12] Carlson BE, Secomb TW. A Theoretical Model for the Myogenic Response Based on the Length—Tension Characteristics of Vascular Smooth Muscle. Microcirculation. 2005; 12: 327–338. 16020079 10.1080/10739680590934745

[13] Collins DM, McCullough WT, Ellsworth ML. Conducted vascular responses: communication across the capillary bed. Microvascular research. 1998; 56: 43–53. 9683562 10.1006/mvre.1998.2076

[14] Arciero JC, Carlson BE, Secomb TW. Theoretical model of metabolic blood flow regulation: roles of ATP release by red blood cells and conducted responses. American Journal of Physiology-Heart and Circulatory Physiology. 2008; 295: 1562–1571. 10.1152/ajpheart.00261.2008 18689501 PMC2593502

[15] Carlson BE, Arciero JC, Secomb TW. Theoretical model of blood flow autoregulation: roles of myogenic, shear-dependent, and metabolic responses. American Journal of Physiology-Heart and Circulatory Physiology. 2008; 295: 1572–1579. 10.1152/ajpheart.00262.2008 18723769 PMC2593503

[16] Cornelissen AJ, Dankelman J, VanBavel E, Spaan JA. Balance between myogenic, flow-dependent, and metabolic flow control in coronary arterial tree: a model study. American Journal of Physiology-Heart and Circulatory Physiology. 2002; 282: 2224–2237. 12003832 10.1152/ajpheart.00491.2001

[17] Liao JC, Kuo L. Interaction between adenosine and flow-induced dilation in coronary microvascular network. American Journal of Physiology-Heart and Circulatory Physiology. 1997; 41: 1571–1581. 10.1152/ajpheart.1997.272.4.H1571 9139938

[18] Kuo L, Davis MJ, Chilian WM. Endothelium-dependent, flow-induced dilation of isolated coronary arterioles. Am J Physiol. 1990; 259: H1063–H1070. 2221113 10.1152/ajpheart.1990.259.4.H1063

[19] Kuo L, Davis MJ, Chilian WM. Longitudinal gradients for endothelium-dependent and-independent vascular responses in the coronary microcirculation. Circulation. 1995; 92: 518–525. 7543382 10.1161/01.cir.92.3.518

[20] Spaan J, Kolyva C, van den Wijngaard J, Ter Wee R, van Horssen P, Piek J, et al Coronary structure and perfusion in health and disease. Philosophical Transactions of the Royal Society A: Mathematical, Physical and Engineering Sciences. 2008; 366: 3137–3153. 10.1098/rsta.2008.0075 18559321

[21] Westerhof N, Stergiopulos N, Noble MM. Law of Laplace In: Snapshots of Hemodynamics: Springer US; 2010 p. 45–48.

[22] Westerhof N, Boer C, Lamberts RR, Sipkema P. Cross-talk between cardiac muscle and coronary vasculature. Physiological Reviews. 2006; 86: 1263–1308. 17015490 10.1152/physrev.00029.2005

[23] Stepp DW, Nishikawa Y, Chilian WM. Regulation of shear stress in the canine coronary microcirculation. Circulation. 1999; 100: 1555–1561. 10510060 10.1161/01.cir.100.14.1555

[24] Pohl U, Lamontagne D. Impaired tissue perfusion after inhibition of endothelium-derived nitric oxide In: Drexler H, Zeiher AM, Bassenge E, Just H, editors. Endothelial Mechanisms of Vasomotor Control: Steinkopff; 1991 p. 97–105. 10.1007/978-3-642-72461-9_11 1953621

[25] Sircar S. Principles of medical physiology: Thieme; 2008.

[26] Mosher P, Ross J, Mcfate PA, Shaw RF. Control of coronary blood flow by an autoregulatory mechanism. Circulation research. 1964; 14: 250–259. 14133952 10.1161/01.res.14.3.250

[27] Jones CJ, Kuo L, Davis MJ, DeFily DV, Chilian WM. Role of nitric oxide in the coronary microvascular responses to adenosine and increased metabolic demand. Circulation. 1995; 91: 1807–1813. 7882491 10.1161/01.cir.91.6.1807

[28] Cohen KD, Berg BR, Sarelius IH. Remote arteriolar dilations in response to muscle contraction under capillaries. American Journal of Physiology-Heart and Circulatory Physiology. 2000; 278: 1916–1923. 10.1152/ajpheart.2000.278.6.H1916 10843889

[29] Mayers I, Johnson DH. Vasodilators do not abolish pulmonary vascular critical closing pressure. Respiration physiology. 1990; 81: 63–73. 2218108 10.1016/0034-5687(90)90070-f

[30] Farhi E, Klocke F, Mates R, Kumar K, Judd R, Canty J, et al Tone-dependent waterfall behavior during venous pressure elevation in isolated canine hearts. Circulation research. 1991; 68: 392–401. 1991345 10.1161/01.res.68.2.392

